# The relationships between lens diameter and ocular biometric parameters: an ultrasound biomicroscopy-based study

**DOI:** 10.3389/fmed.2023.1306276

**Published:** 2024-01-15

**Authors:** Zhiqian Huang, Jiao Qi, Kaiwen Cheng, Shuyu Liu, Keke Zhang, Yu Du, Yi Lu, Xiangjia Zhu

**Affiliations:** ^1^Department of Ophthalmology, Eye and Ear, Nose and Throat Hospital of Fudan University, Shanghai, China; ^2^Eye Institute, Eye and Ear, Nose and Throat Hospital of Fudan University, Shanghai, China; ^3^NHC Key Laboratory of Myopia (Fudan University), Key Laboratory of Myopia, Chinese Academy of Medical Sciences, Shanghai, China; ^4^Shanghai Key Laboratory of Visual Impairment and Restoration, Shanghai, China; ^5^State Key Laboratory of Medical Neurobiology, Shanghai, China

**Keywords:** lens diameter, ultrasound biomicroscopy, white-to-white distance, anterior segment length, axial length

## Abstract

**Purpose:**

This study aims to explore the relationships between lens diameter (LD) measured with ultrasound biomicroscopy (UBM) and ocular biometric parameters.

**Methods:**

Ocular biometric parameters including axial length (AL), white-to-white distance (WTW), anterior chamber depth (ACD), lens thickness (LT) and anterior segment length (ASL) were measured with IOL-Master 700, and the direct measurement of LD was conducted through UBM (ArcScan Insight 100). Relationships between LD and ocular biometric parameters were then investigated. Eyes with AL ≥ 28 mm were defined as eyes with extreme myopia, and eyes with AL < 28 mm were defined as eyes without extreme myopia.

**Results:**

A total of 194 eyes from 194 subjects were included. The mean LD was 9.58 ± 0.49 mm, ranging from 8.60 to 10.96 mm. According to univariate analysis, larger LD was associated with elder age, male gender, larger WTW, ACD and ASL (all *p* < 0.05). Meanwhile, the LD was positively correlated with AL in eyes without extreme myopia (p < 0.05), but not in eyes with extreme myopia (*p* > 0.05). Backward stepwise regressions revealed that a larger LD was associated with larger WTW, ASL and AL in eyes without extreme myopia (all *p* < 0.05), while ASL was the only significant variable in eyes with extreme myopia (p < 0.05).

**Conclusion:**

Larger WTW, ASL and AL in eyes without extreme myopia, as well as longer ASL in eyes with extreme myopia indicated a larger LD, which provides guidance in personalized surgical choice and promises ideal visual outcomes.

## Introduction

Cataract has always been one of the most common causes of vision loss worldwide (1). In recent decades, with the rapid development of accurate biometric techniques and application of various functional intraocular lenses (IOLs), cataract surgery has become more of a refractive surgery aiming at better visual quality, rather than solely visual acuity restorations (2, 3).

However, postoperative misalignment of intraocular lenses (IOLs), encompassing tilt, decentration, and rotation, notably compromises refractive cataract surgery outcomes, particularly with multifocal and toric IOLs (4, 5). It is widely acknowledged that the size of the capsular bag plays a vital role in the postoperative IOL position (3, 6), but the difficulty in measuring lens diameter (LD) directly, as an indicator for the size of the capsular bag, presents challenges for surgeons in choosing a corresponding compatible IOL. Thus, investigation of the relationships of LD with measurable ocular biometric parameters might provide useful information for cataract surgeons to predict LD and further select compatible IOLs.

However, associations between LD and biometric parameters remain controversial due to technique restrictions. Previously, several studies have focused on the measurements of human cadaver eyes and found that the LD was positively correlated with the axial length (AL) and age, but could not be predicted by white-to-white (WTW) distance (7, 8). The ocular magnetic resonance imaging (MRI) was applied to find that the transverse diameter of the eyeball and AL resulted in the best prediction for LD (9), while the results of optical coherence tomography (OCT) showed poor prediction performance (10). Nevertheless, postmortem changes of cadaver lens largely reduced the accuracy, and the sample sizes were limited due to the difficulty in obtaining cadaver lens, and the poor resolution of MRI and the incomplete lens imaging of OCT impaired the reliability of these measurements.

Here we introduced a newly developed very high frequency (VHF) ultrasound biomicroscopy (UBM), ArcScan Insight 100. Compared with conventional UBM, it offers superior signal penetration and better ability to capture the entire anterior segment in a single image, and is less invasive by using a disposable eyepiece, which allows to measure the LD more accurately and conveniently (11–13).

Therefore, in this study, we aimed to investigate the relationships between LD measured with UBM and ocular biometric parameters, thereby guiding surgeons to choose a compatible IOL, and helping patients obtain better visual outcomes.

## Materials and methods

### Ethics statement

The cross-sectional study was performed in accordance with the tenets of the Declaration of Helsinki, approved by the Institutional Review Board of the Eye & ENT Hospital of Fudan University, Shanghai, China, and registered at http://www.clinicaltrials.gov (accession number NCT02182921, protocol number 20201001–1, date of approval December 22nd, 2020). All participants provided signed informed consent, after receiving a full description of the study, for the use of their clinical data.

### Participants

Participants planned to have cataract surgery at Eye & ENT Hospital of Fudan University, Shanghai, China and healthy volunteers were enrolled in this study from January 2022 to December 2022. Exclusion criteria are listed as follows: (1) abnormalities in the position and shape of the lens, such as lens dislocation or subluxation, lens coloboma, etc.; (2) mature or hyper-mature stage of cataract (LOCS III grading: NO score ≥ 5 or NC score ≥ 5 or C score ≥ 4) that impacts the relationships between LD and other ocular parameters; (3) history of strabismus, nystagmus or severe retinal pathologies that affect fixation; (4) history of intraocular surgeries or ocular trauma; (5) unclear UBM images of the anterior segment. One eye was randomly selected from each patient to avoid double-organ bias.

### Ocular biometric measurements by IOL master

Ocular biometric parameters were obtained using the IOL Master 700 (Carl Zeiss AG, Jena, Germany), a non-invasive optical biometer that uses partial coherence interferometry, by experienced technicians. Before measurement, the correct fixation of the examinees was visually checked on the fovea scan by the technician. During each examination, AL, WTW; anterior chamber depth (ACD), and lens thickness (LT) were measured automatically. Meanwhile, the standard deviation (SD) for AL, ACD and LT were also automatically calculated, and the device would warn of low-quality results if the SD for AL >0.027 mm, for ACD >0.021 mm or LT >0.038 mm, which would further be deleted and repeated until reproducible readings were obtained. The anterior segment length (ASL) was calculated by the sum of LT and ACD. Eyes with AL ≥ 28 mm were defined as eyes with extreme myopia, and eyes with AL < 28 mm were defined as eyes without extreme myopia.

### Lens diameter measurement by ultrasound biomicroscopy

**Figure 1:**
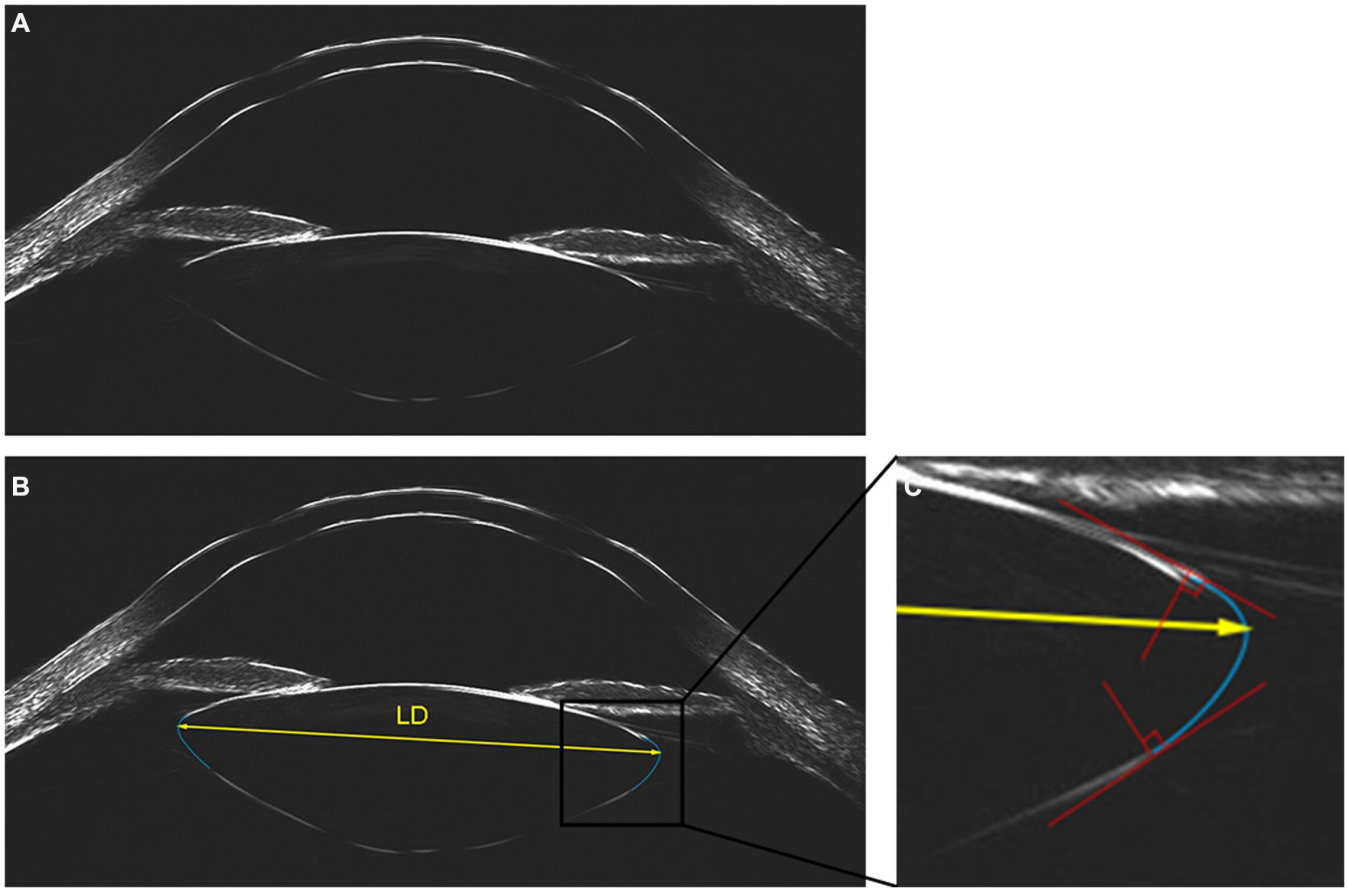
Lens imaging and measurement of lens diameter with ArcScan Insight 100. **(A)** Original image. **(B)** Two best fitting arcs (in blue) were generated to delineate the equator of the lens, then the lens diameter (LD) was measured as the distance between the vertex of the circular arcs on both sides of the lens (in yellow). **(C)** Two auxiliary lines (in red) were introduced at the intersection of our fitting arc and the observed anterior or posterior curve to ensure the alignment, with the perpendicular angle defining the fitting arc (in blue) as the best fit.

Cross-sectional views of the lens with anterior segment imaging were obtained using UBM measurements (Insight 100, ArcScan Inc., Golden, CO, USA), which is a newly developed very high frequency (VHF) ultrasound device for imaging and obtaining biometric measurements of the eye. It uses precision high-frequency ultrasound technology to offer superior signal penetration and to image the true anatomy of the entire anterior segment in a single image with the high resolution at an ultrasonic frequency of 20–60 MHz, a maximum arc scanning range of 80°, and a linear scanning range of 28 mm (Figure 1A). During scanning, seated subjects placed their chin into a headrest and the fellow eye into a soft and rimmed eye-cup, which was then filled with the balanced salt solution. A soft membrane separated the eye from the transducer in a scanning chamber filled with distilled water. With participants staring at a narrow fixation target, the examiner adjusted the scanning frame to be centered on the corneal reflex. The UBM measurement was then performed in the ‘capsule’ mode, with a tissue penetration depth of 15 mm on the axial horizontal section (transverse diameter passing through the corneal apex from 9 to 3 o’clock).

Using the built-in tool of Insight 100, the outline of the lens was delineated and LD was measured by two experienced ophthalmologists independently. The built-in manual caliper tool can generate a fitting arc through three points, which could be adjusted via moving these three points for the best fit. Two auxiliary lines (Figure 1C, depicted in red) were introduced at the intersection of our fitting arc and the observed anterior or posterior curve, which were strategically positioned to ensure that the fitting arc tangentially aligned with the observed curve. The perpendicular orientation of these lines determined the ideal positioning, defining the fitting arc as the best fit (Figure 1C, depicted in blue). Two best fitting arcs were generated to delineate the equator of the lens, effectively outlining its elliptical shape. LD was quantified as the distance between the vertex of the circular arcs on both sides of the lens (Figure 1B, depicted in yellow). The Bland–Altman analysis of two observers showed excellent interobserver reproducibility (Supplementary Figure S1), and the averaged measurements from both observers were utilized for subsequent analyses.

### Statistical analysis

Quantitative data were presented as means ± standard deviations (SD). After assessing the normal distribution of the data with Shapiro–Wilk normality test, comparisons between two groups were performed using Student’s t-test, and among more than two groups using one-way analysis of variance (ANOVA). Categorical data were expressed as the frequencies and percentages of each category, and compared using X^2^ test. The Pearson coefficient was determined to evaluate the strength of all correlation pairs. Multiple linear regressions were performed using backward stepwise selection. Statistical analyses were performed with SPSS version 26.0 (IBM Inc., Chicago, IL, USA) and graphs were prepared using Prism 8.0 (GraphPad Software, Inc., USA). *p* values <0.05 were considered statistically significant.

## Results

### Characteristics

Table 1 presents the demographic and ocular biometric parameters of all participants in this study. This study included 194 eyes of 194 patients (91 men and 103 women). The mean age was 59.2 ± 16.1, ranging from 22 to 90 years old. The study population included 20 (10.31%) eyes with AL < 22 mm, 80 (41.24%) eyes with AL between 22 and 24.5 mm, 28 (14.43%) eyes with AL between 24.5 and 26 mm, 32 (16.50%) eyes with AL between 26 and 28 mm and 34 (17.53%) eyes with AL ≥ 28 mm.

**Table 1:** Demographic and ocular biometric data of all participants.

Parameters	Value
Eyes (n)	194
Age (years)	59.2 ± 16.1 (22–90)
Gender (Male/Female)	91 (46.9%) / 103 (53.1%)
WTW (mm)	11.62 ± 0.50 (10.1–12.7)
ACD (mm)	3.18 ± 0.48 (1.75–4.60)
LT (mm)	4.31 ± 0.52 (3.02–5.87)
ASL (mm)	7.49 ± 0.44 (5.99–9.09)
AL (mm)	25.39 ± 3.11 (21.07–35.33)

WTW, white-to-white distance; ACD, anterior chamber depth; LT, lens thickness; ASL, anterior segment length; AL, axial length.

Data were presented as mean ± standard deviation (range or proportion).

**Figure 2:**
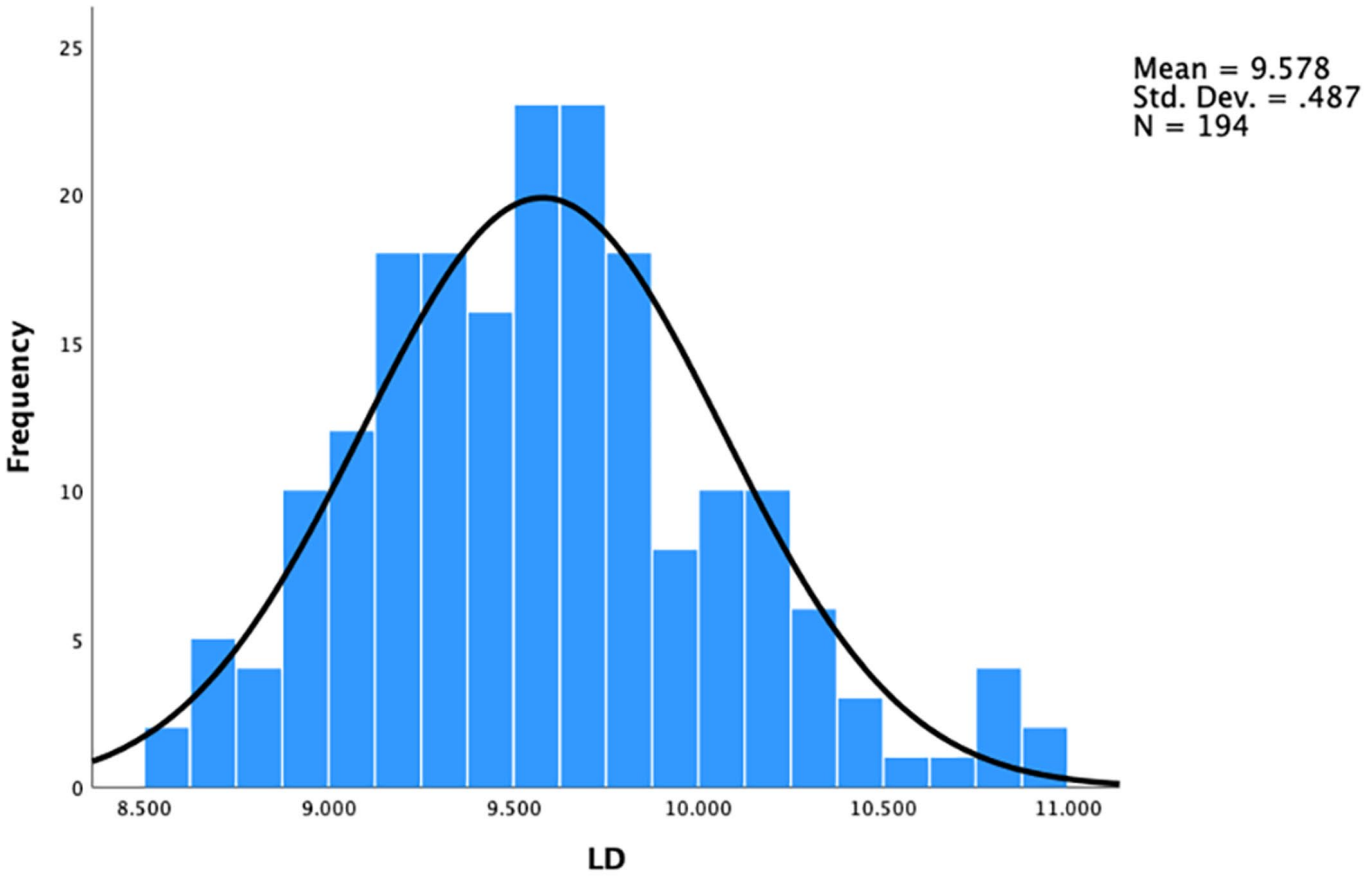
Distribution of lens diameter (LD) among this study population.

The distribution of LD among this study population is presented in Figure 2. The mean LD was 9.58 ± 0.49 mm, ranging from 8.60 to 10.96 mm. The 25 and 75% percentiles of LD were 9.22 and 9.84, respectively.

### Univariate analysis of the correlations between lens diameter and other variables

**Figure 3:**
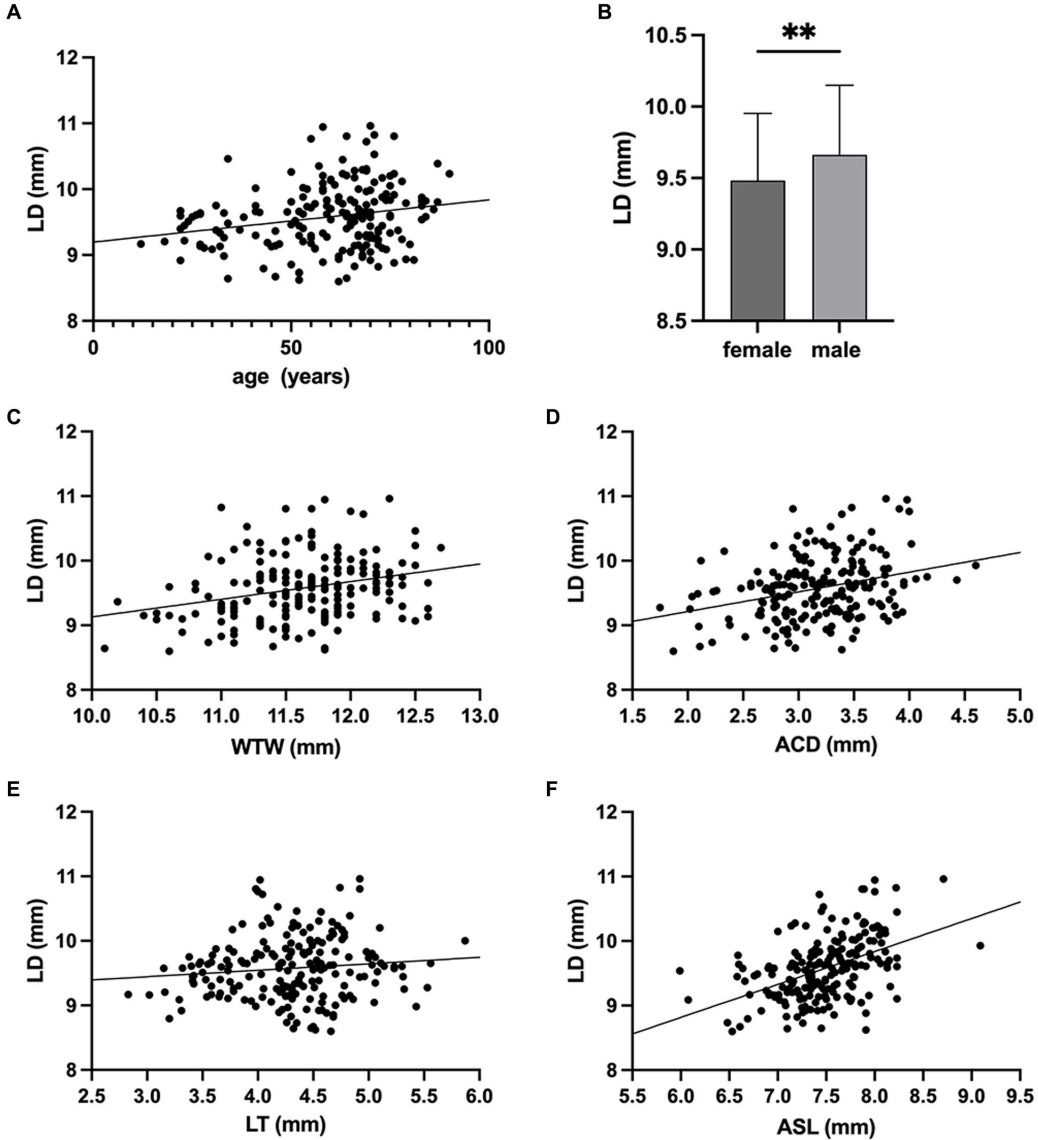
Associations of lens diameter (LD) with age, sex and ocular biometric parameters. **(A)** LD was positively correlated with age (*r* = 0.206, *p* = 0.004). **(B)** The LD of males was significantly larger than that of females (** *p* < 0.01). Correlations between lens diameter (LD) and **(C)** white-to-white distance (WTW) (*r* = 0.288, *p* < 0.001), **(D)** anterior chamber depth (ACD) (*r* = 0.331, *p* < 0.001), **(E)** lens thickness (LT) (*r* = 0.074, *p* = 0.301), **(F)** anterior segment length (ASL) (*r* = 0.448, *p* < 0.001).

The LD was positively correlated with age (Pearson’s correlation coefficient, *r* = 0.206, *p* = 0.004; Figure 3A), and the male subjects had significantly larger LD than female subjects (9.68 ± 0.48 mm for males and 9.49 ± 0.48 mm for females, *p* = 0.009, Figure 3B). As presented in the scatterplots for LD against WTW, ACD, LT, and ASL, the Pearson’s correlation analysis revealed that LD was positively correlated with WTW (*r* = 0.288, *p* < 0.001; Figure 3C), ACD (*r* = 0.331, *p* < 0.001; Figure 3D), ASL (*r* = 0.448, *p* < 0.001; Figure 3F), whereas, no correlation was found between LD and LT (*r* = 0.074, *p* = 0.301; Figure 3E).

**Figure 4:**
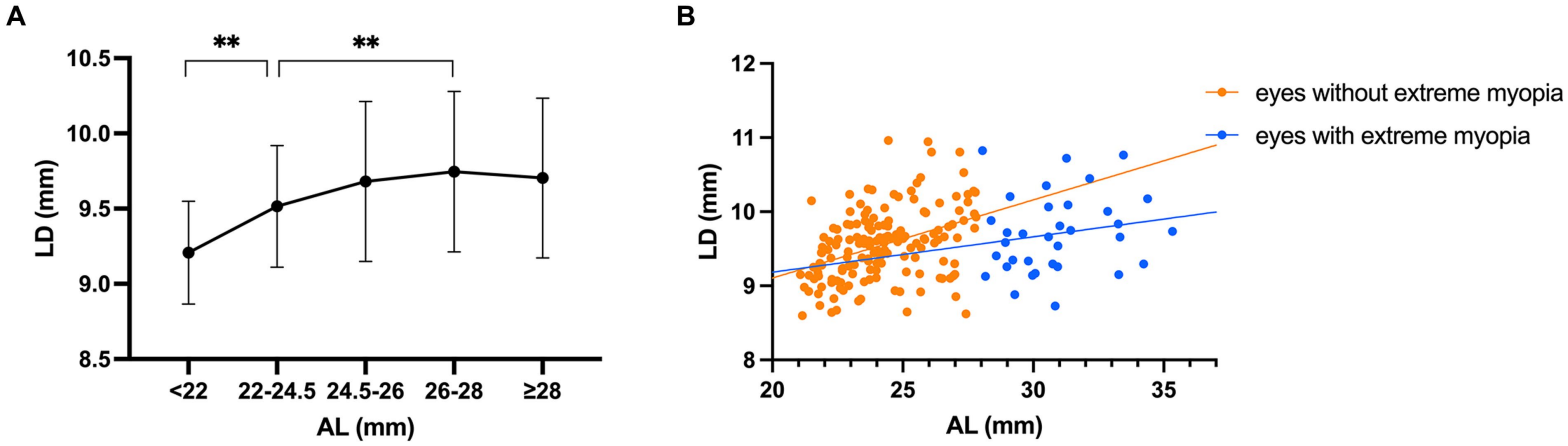
Changes of lens diameter (LD) with axial length (AL). **(A)** Compared with the AL 22–24.5 mm group, the AL < 22 mm group had significantly smaller LDs and the AL 26–28 mm group had significantly larger LDs (** *p* < 0.01). With the increase of AL, the LD was firstly increased gradually to peak in the AL 26–28 mm group, then decreased slightly. **(B)** The correlation between LD and AL was significantly positive in eyes without extreme myopia (*r* = 0.398, *p* < 0.001), but not significant in eyes with extreme myopia (*r* = 0.175, *p* = 0.323).

Change of LD with AL is presented in Figure 4A. With the increase of AL, the LD was firstly increased gradually to peak in the group with AL of 26-28 mm, then decreased slightly (ANOVA, *p* < 0.05). Pearson’s correlation analysis showed that the LD correlated positively with AL in eyes without extreme myopia (*r* = 0.398, *p* < 0.001; Figure 4B), but not correlated in eyes with extreme myopia (*r* = 0.175, *p* = 0.323; Figure 4B).

### Independent predictive factors of lens diameter with multivariate analysis

Backward stepwise multiple linear regressions, which included age, sex, WTW, ASL and AL as independent variables, were performed to evaluate the independent predictors of LD in eyes without and with extreme myopia, respectively. As shown in Table 2, after adjusting for age and sex, a larger LD was associated with larger WTW (β = 0.165, *p* = 0.019), longer ASL (β = 0.232, *p* = 0.010) and longer AL (β = 0.074, *p* = 0.001) in eyes without extreme myopia. Whereas, in eyes with extreme myopia, only ASL (β = 0.693, *p* = 0.014) was independently correlated with LD.

**Table 2:** Multiple linear regressions analysis of LD in eyes without and with extreme myopia.

	**Variables**	**β Coefficient**	**Std. Error**	***P* value**
Eyes without extreme myopia	WTW	0.165	0.069	0.019
	ASL	0.232	0.089	0.010
	AL	0.074	0.021	0.001
Eyes with extreme myopia	ASL	0.693	0.268	0.014

WTW, white-to-white distance; ASL, anterior segment length; AL, axial length.

## Discussion

Cataract is a common ocular disease which can cause vision impairment and seriously affect the patient’s quality of life. With advances in surgical technology and application of various functional IOLs, cataract surgery has entered the era of refractive surgery, which can not only rebuild vision but also provide good visual quality. The postoperative stability of the IOL position is the critical fundament of achieving optimal visual outcomes, with the match between the capsular bag and IOL serving as the determinant (2, 3, 5). However, whether the regular biometry could predict the LD has not yet been reached, and the best approach to measure or calculate the LD was also sought for (14–16). In this study, we used a novel UBM to measure LD and investigate the relationships between the LD and ocular biometric parameters, and observed that a larger LD was associated with larger WTW, ASL and AL in eyes without extreme myopia, while ASL was the only significant predictor in eyes with extreme myopia.

In our study, we firstly used a novel UBM to measure LD and investigate its predictors. Revolving around LD, early efforts were based on *ex vivo* measurements of human cadaver eyes (7, 8). However, due to the postmortem changes of cadaver lens, the deviation from its physiological shape should not be overlooked. And since the lens shape was dependent on the accommodation force, the extracted lens without any accommodation was not comparable to the lens *in vivo* (17). Compared with previous studies based on cadaver eyes, our study avoided the postmortem changes of cadaver lens from its physiological shape, rendering our findings more precise and compelling (12, 13). Besides, the primitive methods of measurement may lack accuracy, and the sample sizes of both studies was relatively small. Also, plenty of *in vivo* measurements have been performed, such as MRI and OCT, but a reliable and easily available approach to predict LD was hardly found. The resolution of MRI remained relatively lower, and given its expensive and inconvenient nature, routine utilization was impractical (9). Regarding the commonly employed measuring methodology based on OCT imaging, the distance between the intersections of the extended anterior and posterior lenticular surface curvatures was regarded as the lens equatorial diameter, which obviously failed to match with the actual elliptical shape of the lens (10, 14, 18, 19). On the contrary, the strength of Insight 100 lies in its ability to detect the peripheral area behind the iris, thus enhancing its capability to depict the real elliptical shape of the lens after the rigorous fitting procedure mentioned above, rather than a spindle-shaped one. As evidenced by recent studies, measurements of lens diameters with Insight 100 were notably shorter compared to those obtained using OCT. This disparity suggests that the enhanced accuracy offered by Insight 100 could carry considerable implications in guiding more informed surgical decisions (12, 13). Besides, compared with conventional UBM, the newly developed Insight 100 exhibits superior signal penetration and reduced invasiveness using a disposable eyepiece, indicating the potential for broader applications (11–13).

In our study, we evaluated the relationships between LD and ocular biometric parameters *in vivo* and found a non-linear correlation between LD and AL. To our best knowledge, our study was the largest sample-size LD prediction study based on ocular biometric parameters. Previously, the conventional idea that longer eyes have a larger capsular diameter was widely believed, as several studies based on cadaver eyes and *in vivo* measurements including OCT and MRI have consistently exhibited a positive association between LD and AL (6, 8–10, 14, 19–21). Contrary to prior expectations, we found that the correlation between LD and AL was not linear. In eyes without extreme myopia, as historically proposed, the LD was on the rise as the AL increased, but that was not the case in eyes with extreme myopia, which could be explained by morphological characteristics of the elongation of myopic eyes. Normally, the eyeball grows globally and uniformly with AL increasing. However, as suggested by our previous finding, extreme elongation of the eyeball was mainly due to the extension of the posterior segment (22). Thus, in eyes with extreme myopia, the LD, as an anterior structure, was not further increased with the eyeball extension.

In our study, we also provided evidence for predicting LD using ocular biometric parameters. We demonstrated that larger AL, WTW and ASL were associated with larger LD in eyes without extreme myopia, and larger ASL was only independent predictor for larger LD in eyes with extreme myopia. The positive correlations of WTW and ASL with LD was consistent with our previous finding that toric IOLs rotate more in eyes with larger WTW and longer ASL (23). One reasonable explanation could be that WTW serves as an indicator of the horizontal diameter of the eyeball, thus representing the horizontal size of the anterior segment to a certain extent (24), and ASL has been established to represent the sagittal dimension of the anterior segment (22, 23). Based on the above findings, we should pay special attention to the selection of an appropriate IOL in the clinical management of patients with these characteristics to avoid postoperative IOL decentration, tilt and rotation.

Several limitations exist in this study. First, the single-hospital setting and exclusion criteria may introduce selection bias, warranting caution when extrapolating findings to broader populations. Second, potential confounding factors, which we may not have fully accounted for, require further elucidation in future research. Third, given the inherent imaging limitations of Insight 100 and the potential deviations introduced by manual measurement, our measuring protocol has not been established as a gold standard for *in vivo* lens biometry, so further explorations into advanced imaging techniques or software tools that offer improved resolution or better delineation of the lens equator still remain a priority.

## Conclusion

To conclude, with the novel UBM introduced, our study demonstrated that a larger LD was associated with elder age, male gender, larger WTW, ACD and ASL, while the correlation between AL and LD was not linear. Conventional parameters including AL, WTW and ASL in eyes without extreme myopia, as well as ASL in eyes with extreme myopia would help better to predict LD, hopefully to aid in personalized surgical decision-making and to promise ideal visual outcomes.

## Data availability statement

The raw data supporting the conclusions of this article will be made available by the authors, without undue reservation.

## Ethics statement

The studies involving humans were approved by the Institutional Review Board of the Eye & ENT Hospital of Fudan University, Shanghai, China. The studies were conducted in accordance with the local legislation and institutional requirements. The participants provided their written informed consent to participate in this study.

## Author contributions

ZH: Conceptualization, Data curation, Formal analysis, Investigation, Writing – original draft. JQ: Data curation, Writing – review & editing. KC: Data curation, Writing – review & editing. SL: Data curation, Writing – review & editing. KZ: Data curation, Writing – review & editing. YD: Data curation, Writing – review & editing. YL: Funding acquisition, Resources, Supervision, Writing – review & editing. XZ: Conceptualization, Funding acquisition, Resources, Supervision, Writing – review & editing.

## Supplementary material

SUPPLEMENTARY FIGURE S1 The Bland-Altman analysis of two observers in measuring LDs showed excellent interobserver reproducibility.
