# Supplementary figures and images for: The relationships between lens diameter and ocular biometric parameters: an ultrasound biomicroscopy-based study
Source: Front Med (Lausanne). 2024 Jan 15;10:1306276. doi: 10.3389/fmed.2023.1306276 (PMC10822951; doi:10.3389/fmed.2023.1306276)

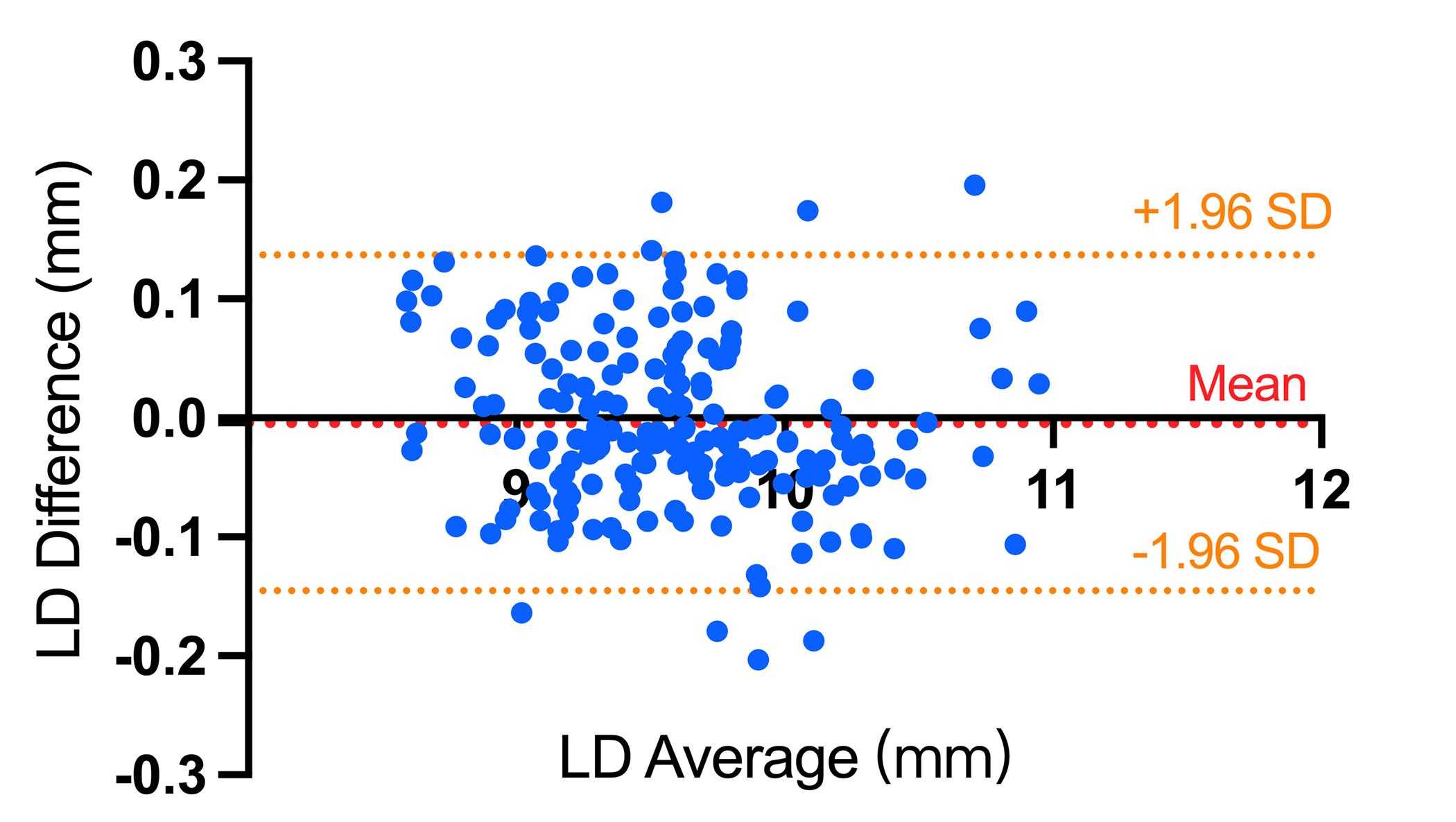

Supplement: SUPPLEMENTARY FIGURE S1 — The Bland-Altman analysis of two observers in measuring LDs showed excellent interobserver reproducibility. [file Image_1.tiff]
